# Gonococcal resistance can be viewed productively as part of a syndemic of antimicrobial resistance: an ecological analysis of 30 European countries

**DOI:** 10.1186/s13756-020-00764-z

**Published:** 2020-06-30

**Authors:** Chris Kenyon, Sheeba S. Manoharan-Basil, Christophe Van Dijck

**Affiliations:** 1 HIV/STI Unit, Institute of Tropical Medicine, 2000 Antwerp, Belgium; 2 Division of Infectious Diseases and HIV Medicine, University of Cape Town, Anzio Road, Observatory, Cape Town, 7700 South Africa

**Keywords:** Gonorrhoea, *Neisseria gonorrhoeae*, Fluoroquinolones, Macrolides, Antimicrobial resistance, Stewardship, Antibiotic consumption, Bystander selection

## Abstract

**Background:**

It is unclear how important bystander selection is in the genesis of antimicrobial resistance (AMR) in *Neisseria gonorrhoeae*.

**Methods:**

We assessed bystander selection in a novel way. Mixed-effects linear regression was used to assess if country-level prevalence of gonococcal AMR in 30 European countries predicts homologous AMR in other bacteria. The data used was from the European Antimicrobial Resistance Surveillance Network.

**Results:**

The prevalence of gonococcal ciprofloxacin resistance was found to be positively associated with AMR prevalence in *E. coli* (coef. 0.52; *P* = 0.007), *Acinetobacter spp*. (coef. 0.13; *P* = 0.044) and *Pseudomonas aeruginosa* (coef. 0.36; *P* = 0.020) but not *Klebsiella pneumoniae*. Azithromycin resistance in *N. gonorrhoeae* was positively associated with macrolide resistance in *Streptococcus pneumoniae* (coef. 0.01; *P* = 0.018). No association was found for cephalosporins.

**Conclusions:**

Gonococcal AMR is linked to that in other bacteria. This finding is likely explained by high antimicrobial consumption in affected populations and provides additional motivation for strengthening antimicrobial stewardship programs.

## Background

**Fig. 1:**
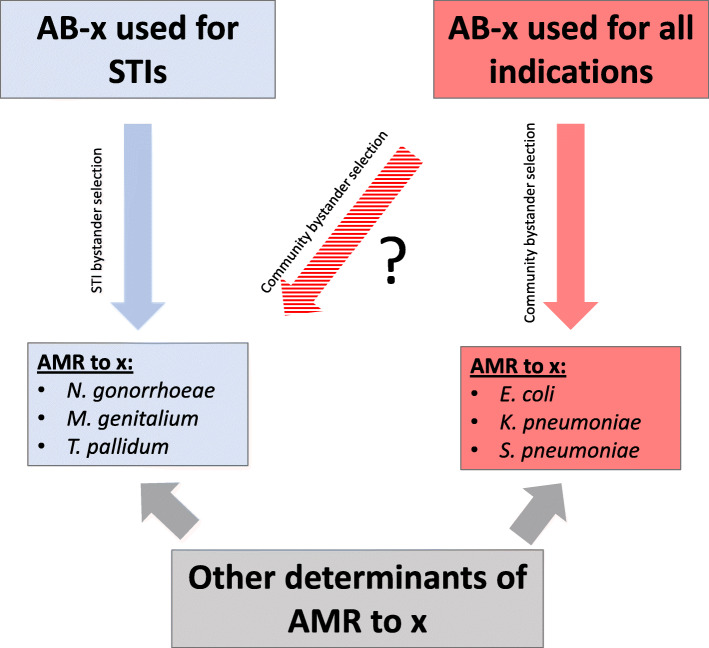
Conceptual framework for understanding how different forms of bystander selection could result in *N. gonorrhoeae* antimicrobial resistance (AMR) to the antibiotic-x (AB-x). The blue-pathway represents STI bystander selection where the use of antibiotic-x to treat STIs selects for AMR to other STIs that were not the target of the antibiotic. The red-pathway depicts community bystander selection whereby high-levels of consumption of antibiotic-x would select for resistance to ‘x’ in circulating commensal and pathogenic bacteria. This community bystander selection could also act on STIs (striped red arrow), one of the hypotheses tested in this paper. The other hypothesis tested is that the prevalence of AMR to ‘x’ in *N. gonorrhoeae* will be positively associated with that in *Escherichia coli* and other pathobionts

*Neisseria gonorrhoeae* has developed antimicrobial resistance (AMR) to every major class of antimicrobials used to treat it [1, 2]. There are real concerns that it may be untreatable with available antimicrobials in the not too distant future [2, 3]. Understanding the determinants of AMR in *N. gonorrhoeae* is vital to prevent the future emergence of AMR. Initially, it was thought that the major AMR determinant was exposure to antibiotics used to treat gonorrhoea [4–6]. The appreciation that *N. gonorrhoeae* is asymptomatic for much or most of the time it circulates in a population means that antibiotics used for other indications needed to be considered (bystander selection) [7]. Since gonococcal infections cluster with other STIs, a widely held formulation of this view was that bystander selection was predominantly confined to antibiotics used to treat other STIs (termed the STI bystander theory) [8]. While authors have speculated that drugs such as macrolides used to treat chlamydial and *Mycoplasma genitalium* genital infections may drive AMR in *N. gonorrhoeae*, little direct evidence to support this notion has been published [9]. One study has, however, found that the prevalence of macrolide AMR in *Treponema pallidum*, the bacterium responsible for syphilis, was strongly associated with population-level consumption of macrolides [10]. Other studies have suggested that antibiotic consumption for all indications plays a role (community bystander theory; Fig. 1) [7]. Understanding which of these theories is correct has important implications. If the former is true, then preventing the further emergence of gonococcal AMR could be accomplished by interventions such as antimicrobial stewardship limited to within STI services. If total antibiotic consumption played a role, then stewardship efforts to reduce antibiotic consumption in the whole community would be important [11].

Ecological studies have reached different conclusions regarding the association between general antimicrobial consumption and the emergence of AMR in *N. gonorrhoeae*, with some studies finding no association [12] and others finding an association [11, 13, 14]. Given the complexity of resistance ecology and the crudeness of the measurement tools at our disposal, these differences in findings are not too surprising [15]. For example, population-level antimicrobial exposure is typically measured as defined daily doses (DDD) per 1000 persons. The effect of exposure on AMR, however, depends on a variety of pharmacokinetic and other variables. Thus, a population that uses a higher and more effective dosing schema of antibiotics may have a higher DDD but a lower probability for inducing AMR to that antibiotic [15]. Exposure in a remote location may also result in AMR, which may then spread via travel to populations without the exposure [16]. This problem is particularly important for *N. gonorrhoeae* where travel has been shown to have played a vital role in the spread of AMR [16]. The problem is further compounded by horizontal gene transfer of AMR-conferring DNA from different bacterial species, where the AMR may persist (and travel) for years following exposure [17–20]. The emergence of AMR may also be due to a short, concentrated period of exposure in a particular subpopulation and the AMR irreversible despite reduced antimicrobial exposure. These features mean that traditional country-level ecological analyses may miss associations between antimicrobial exposure and resistance.

These limitations motivated the current study where we approach the problem via a novel methodology. In addition to assessing the traditional association between antimicrobial consumption and gonococcal AMR, we analyze the association between gonococcal AMR and homologous AMR in other pathobionts. The hypothesis we test is that gonococcal AMR is associated with AMR in these pathobionts (Fig. 1). Finding evidence of such an association, we argue that this is most parsimoniously explained by all these bacteria being exposed to high levels of the respective antimicrobial for all indications.

## Methods

### Data

#### Antimicrobial resistance data

The AMR data was taken from the European Centre for Disease Prevention and Control (ECDC) Surveillance Atlas which reports resistance prevalence estimates from the European Antimicrobial Resistance Surveillance Network (EARS-Net) - the EU’s main surveillance system for AMR in bacteria that cause serious infections. All 28 EU Member States and two EEA countries (Iceland and Norway) participate in EARS-Net. The countries provide data for all eight species under surveillance (*Escherichia coli, Klebsiella pneumoniae, Streptococcus pneumoniae, Acinetobacter spp., Pseudomonas aeruginosa, Enterococcus faecalis, Enterococcus faecium, Staphylococcus aureus*), with the exception of Greece which did not report data on *S. pneumoniae.* Only data from invasive (blood and cerebrospinal fluid) isolates are included in EARS-Net. This is done to limit biases that may emerge if isolates from all anatomical sites were included. Further details can be found in the EARS-Net reporting protocol [21]. The system depends on national network representatives in each participating country, reporting their locally tested susceptibility data to The European Surveillance System on an annual basis. This data is available for public access at https://www.ecdc.europa.eu/en/surveillance-atlas-infectious-diseases

The *N. gonorrhoeae* AMR surveillance data was extracted from the same ECDC Surveillance Atlas. This data comes from the Euro-Gonococcal Antimicrobial Surveillance Programme which uses a different methodology and includes a sentinel AMR surveillance program that tests a representative number of isolates from EU/EEA member states every year for a range of antimicrobials, through a hybrid centralized/decentralized system [22, 23]. Data is available from 2000 (or later) to 2018.

The ECDC Surveillance Atlas reports gonococcal AMR for ciprofloxacin (a fluoroquinolone), azithromycin (a macrolide), cefixime and ceftriaxone (both Extended Spectrum (ES) cephalosporins) by country and year for 30 countries from 1997 to 2018. Since our hypothesis tested homologous class bystander selection, we limited our analysis to the 5 bacterial species that reported AMR for fluoroquinolones, macrolides or ESCephalosporins (*E. coli, K. pneumoniae, S. pneumoniae, Acinetobacter spp., P. aeruginosa*). The following minimum inhibitory concentration (MIC) breakpoints were used to define gonococcal antimicrobial resistance: Azithromycin: > 0.5 mg/L, Cefixime: > 0.12 mg/L, Ceftriaxone: > 0.12 mg/L, Ciprofloxacin: > 0.06 mg/L [1, 24]. The breakpoints used for the other species are detailed elsewhere [22, 23]. We use the term ‘antimicrobial resistance’ in its broadest sense to include reduced susceptibility.

### Antimicrobial consumption data

Data from the European Surveillance of Antimicrobial Consumption (ESAC) were used as a measure of national general population-level antimicrobial drug consumption [25, 26]. ESAC provides open access to the data collected on antimicrobial use in ambulatory care and hospital care in 30 European countries [25, 26]. ESAC reports antimicrobial consumption as the number of defined daily doses (DDD) per 1000 inhabitants following the World Health Organization guidelines [26, 27]. One DDD is defined as the average maintenance dose per day for a drug used in its main indication for adults [26]. In our study, we used three measures of country-specific antimicrobial drug use in ambulatory care: Cephalosporins/other Beta lactams (ATC group J01D), fluoroquinolones (ATC group J01MA), macrolides, lincosamides and streptogramins (ATC group J01F). Data was available from 1998 to 2018.

### Analyses

#### Associations between AMR in N. gonorrhoeae and other species

For each antibiotic class, mixed effects linear regression was used to assess the association between the prevalence of AMR in *N. gonorrhoeae* and each of the other bacterial species. The following mixed effects linear model was used:

(*N.gonorrhoeae*_resistance-to-X in year Y and country C) ~ (*Species-Z*_resistance-to-X in year Y and country C) + (random intercept for country C) + intercept + error, where species-Z is one of *E. coli, K. pneumoniae, S. pneumoniae, Acinetobacter spp. or P. aeruginosa,* and X could be fluoroquinolones, macrolides or ESCephalosporins.

**Fig. 2:**
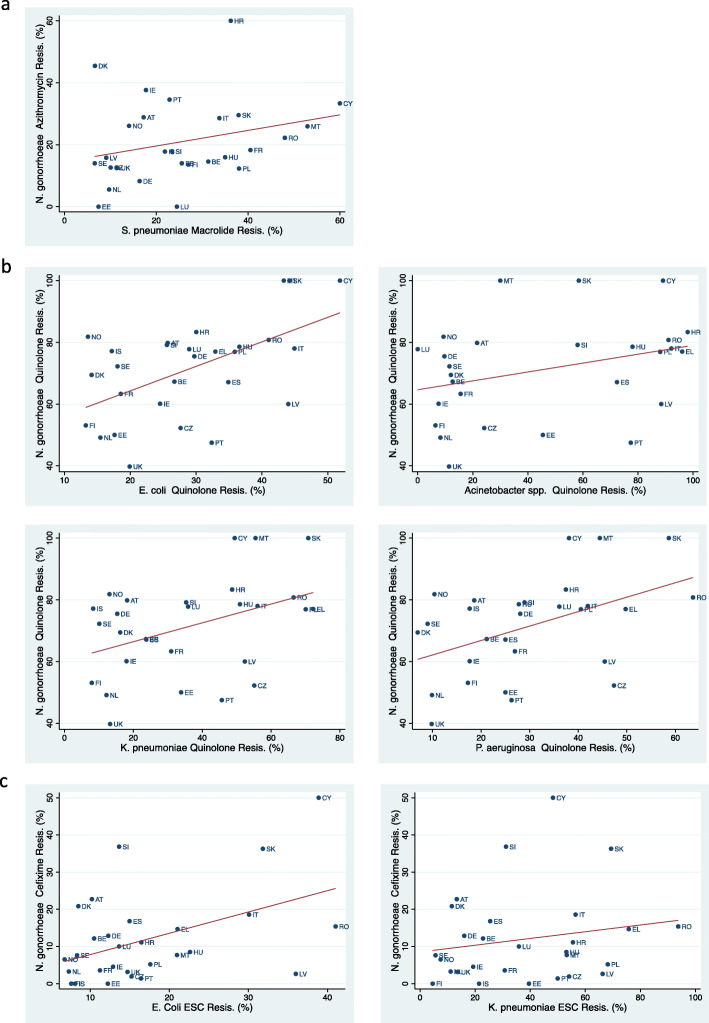
Association between peak antimicrobial resistance prevalence (2000 to 2018) in *Neisseria gonorrhoea* and other bacteria for (**a**) Macrolides - *Streptococcus pneumoniae*, (**b**) Fluoroquinolones. - *Escherichia coli, Klebsiella pneumoniae, Pseudomonas aeruginosa, Acinetobacter spp*. and (**c**) Extended Spectrum Cephalosporins (ESC) - *Escherichia coli* and *Klebsiella pneumoniae*

We represented the visual associations in AMR between *N. gonorrhoeae* and the other species with scatterplots using peak AMR prevalence, which was defined as the maximum AMR prevalence attained in each country for the period under observation. The ‘maximum AMR prevalence’ variable was only used in the generation of Fig. 2.

#### Associations between AMR and antimicrobial consumption

In separate analyses for each relevant antimicrobial-species combination, mixed effects linear regression was used to assess the correlation between prevalence of AMR and antimicrobial consumption in the preceding year. The following mixed effects linear model was used:

(MIC/resistance in year Y and country C) ~ (antimicrobial consumption in year Y-1 and country C) + (random intercept for country C) + intercept + error.

The statistical analyses were performed in Stata 16.0. A *P*-value of < 0.05 was regarded as significant.

### Ethics approval

This analysis involved ecological analyses of public access data and thus no Ethics approval was necessary.

## Results

There were large variations (up to 300-fold) in the consumption of cephalosporins, fluoroquinolones and macrolides between countries (Table 1, Figures S1-S2). The median consumption of each class of antibiotic did not change by more than 20% between 1997 and 2018.

**Table 1:** Variation in antimicrobial consumption and resistance to cephalosporins, fluoroquinolones and macrolides for six bacterial species in 30 European Countries. Values reported as medians (interquartile ranges)

	Antimicrobial consumption				Antimicrobial Resistance											
					*Neisseria gonorrhoeae*		*Escherichia coli*		*Klebsiella pneumoniae*		*Streptococcus pneumoniae*		*Acinetobacter spp.*		*Pseudomonas aeruginosa*	
	1997	FD	2018	FD	2009	2018	2000	2018	2005	2018	2005	2018	2005	2018	2005	2018
N	14		29		17	27	4	29	21	29	26	28	26	28	26	29
ESCephalosporin	1.7 (0.6–4.0)	313	1.9 (0.6–2.7)	262	0.9 (0.0–6.4)^a^	0.0 (0.0–2.2)^a^	0.2 (0.1–1.9)	13.8 (9.6–19.3)	7.1 (4.1–27.7)	30.8 (12.8–53.3)	NA	NA	NA	NA	NA	NA
Fluoroquinolone	1.0 (0.5–1.6)	13.8	1.2 (0.8–2.3)	7.0	70.0 (49.1–79.2)	55.0 (44.4–60.0)	3.8 (2.5–6.0)	23.9 (17.7–32.1)	11.1 (4.9–34.0)	30.4 (13.2–52.7)	NA	NA	15.2 (8.1–22.5)	13.3 (7.5–20.5)	15.2 (8.1–22.5)	15.0 (10.4–26.0)
Macrolide	2.9 (1.8–3.4)	5.4	2.8 (1.9–3.6)	12.7	6.6 (2.6–14.5)	7.7 (3.2–12.7)	NA	NA	NA	NA	15.2 (8.1–22.5)	13.3 (7.5–20.5)	NA	NA	NA	NA

^a^ Cefixime; *ESCephalosporin* Extended Spectrum Cephalosporin, *FD* Fold Difference in antimicrobial consumption between highest and lowest consumption country-year, *NA* Not Available/Not Applicable

For each of the bacterial species, the prevalence of AMR varied considerably between countries (Table 1, Figures S1-S2). There were marked increases in ESCephalosporin and fluoroquinolone resistance over time in both *E. coli* and *K. pneumoniae.* The prevalence of fluoroquinolone resistance in *N. gonorrhoeae* declined over time.

### Association between Ng AMR and other bacteria

#### Fluoroquinolones

The prevalence of gonococcal AMR was positively associated with AMR prevalence in *E. coli* (coef. 0.52; *P* = 0.007), *Acinetobacter spp*. (coef. 0.13; *P* = 0.044) and *P. aeruginosa* (coef. 0.36; *P* = 0.020) but not *K. pneumoniae* (Table 2; Fig. 2).

**Table 2:** Mixed-Effects Linear Regression Analyses of the Relationship Between Antimicrobial Resistance (AMR) in *N. gonorrhoeae* with other pathobionts in 30 European countries

	Azithromycin		Ciprofloxacin		Cefixime	
	Coeff. ± SE	*P*	Coeff. ± SE	*P*	Coeff. ± SE	*P*
*Escherichia coli*	NA		0.52 ± 0.19	0.007	−0.005 ± 0.075	0.948
*Klebsiella pneumoniae*	NA		0.01 ± 0.10	0.943	0.02 ± 0.03	0.503
*Streptococcus pneumoniae*	0.01 ± 0.004	0.018	NA		NA	
*Acinetobacter spp.*	NA		0.13 ± 0.06	0.044	NA	
*Pseudomonas aeruginosa*	NA		0.36 ± 0.15	0.020	NA	

*NA* Not Available/Not Applicable, *SE* Standard Error

#### Macrolides

Azithromycin resistance in *N. gonorrhoeae* was positively associated with macrolide resistance in *S. pneumoniae* (coef. 0.01; *P* = 0.018; Table 2; Fig. 2).

#### ESCephalosporins

No significant associations were found (Table 2; Fig. 2).

### Association between AMR and antimicrobial consumption

#### Fluoroquinolones

The consumption of fluoroquinolones was positively associated with AMR prevalence in *E. coli* (coef. 6.13; *P* < 0.001), *Acinetobacter spp*. (coef. 4.8; *P* < 0.001), *N. gonorrhoeae* (coef. 3.9; *P* = 0.047) and *K. pneumoniae* (coef. 5.4; *P* < 0.001) but not *P. aeruginosa* (Table 3).

**Table 3:** Mixed-Effects Linear Regression Analyses of the Relationship Between Antimicrobial Resistance (AMR) and homologous class Antimicrobial Consumption Among Select Pathobionts in 30 European countries

	Azithromycin		Ciprofloxacin		ESC/Cefixime	
	Coeff. ± SE	*P*	Coeff. ±SE	*P*	Coeff. ± SE	*P*
*Escherichia coli*	NA		6.13 ± 0.60	< 0.001	0.52 ± 0.155	0.155
*Klebsiella pneumoniae*	NA		5.44 ± 1.20	< 0.001	−0.08 ± 077	0.910
*Streptococcus pneumoniae*	2.39 ± 0.61	< 0.001	NA		NA	
*Acinetobacter spp.*	NA		4.75 ± 1.34	< 0.001	NA	
*Pseudomonas aeruginosa*	NA		0.34 ± 0.82	0.676	NA	
*Neisseria gonorrhoeae*	0.52 ± 0.40	0.181	3.86 ± 1.94	0.047	0.49 ± 0.29	0.095

*NA* Not Available/Not Applicable, *ESC* Extended Spectrum Cephalosporins, *SE* Standard Error

#### Macrolides

Macrolide resistance in *S. pneumoniae* (coef. 2.39; *P* < 0.001), but not *N. gonorrhoeae* was associated with macrolide consumption (Table 3).

#### ESCephalosporins

No significant associations were found (Table 3).

## Discussion

We found that the prevalence of gono- and pneumococcal resistance to macrolides was positively associated. Likewise, gonococcal fluoroquinolone resistance was associated with homologous resistance in three of the 4 gram-negative bacteria assessed. No associations with ESCephalosporin resistance were found. In keeping with a previous analysis of European data, only fluoroquinolone consumption was found to be associated with homologous gonococcal resistance [13]. Fluoroquinolone AMR was significantly associated with consumption for all four of the other bacteria assessed. These findings suggest that fluoroquinolone consumption in the general population (community bystander selection), is a parsimonious explanation for variations in fluoroquinolone resistance in these bacteria, including *N. gonorrhoeae.* Of note, these associations were strongest with *E. coli* which is unsurprising if we recall that *E. coli* is the most prevalent of all the bacteria considered in the general human population [7]. This feature would result in it being more exposed to antimicrobial selection pressure in high consumption populations than lower prevalence bacteria [7, 28]. Studies from Europe and elsewhere have found community level consumption of antibiotics such as fluoroquinolones to be strongly associated with AMR in *E. coli* [29]. A weaker or no association has been found with bacteria whose prevalence is lower and where the nosocomial acquisition of AMR is more important such as *K. pneumoniae* [28].

Since 2012, the prevalence of macrolide resistance in *N. gonorrhoeae* has been slowly increasing in Europe [23]. By 2018, the prevalence of azithromycin resistance exceeded 10% in 10 countries (Figure S2). European treatment guidelines have recommended azithromycin together with ceftriaxone as dual therapy for gonorrhoea since 2012 [30], which represents one possible explanation for this increase [31]. This should not, however, explain the increase in AMR in certain European countries but not others. No individual or ecological-level study that we are aware of has found an association between dual therapy and azithromycin reduced susceptibility. European guidelines for non-gonococcal urethritis have strongly advocated doxycycline over azithromycin to prevent bystander selection for macrolide resistance selection in other STIs [32]. This should have lessened the STI bystander selection pressure. Our finding that gonococcal macrolide resistance is weakly associated with resistance in *S. pneumoniae* suggests that community-level macrolide consumption may be playing a role. This may sound odd considering we found no statistically significant association between macrolide consumption and resistance in *N. gonorrhoeae*. Macrolide exposure is, however, a well-established determinant of AMR in both species [14, 33] and was associated with resistance in *S. pneumoniae*. The considerably lower prevalence of gono- compared to pneumococcus as well as biological differences between the organisms may explain the weaker association between consumption and resistance in *N. gonorrhoea.* There are significant parallels between the pathways to macrolide resistance in these two bacteria. In both, resistance is mediated by 23S rRNA target modification and enhanced efflux, conferring high- and low-level resistance, respectively [19, 34, 35]. Furthermore, in both, horizontal gene transfer (via transformation or transposons) from commensal oro- and naso-pharyngeal *Streptococci/Neisseria* has been shown to play an important role in macrolide resistance via uptake of DNA that results in enhanced efflux of macrolides [19, 35, 36]. A single dose of a macrolide can result in long term (over 6-month) elevations in the proportion of commensal *Streptococci* [37] and possibly commensal *Neisseria* [18, 38] with macrolide resistance. This means that a gono- or pneumococcal pharyngeal infection many months after a dose of macrolides may still be able to take up the resistance-conferring DNA from commensals. This delayed effect between antimicrobial consumption and resistance may make it more difficult to detect in traditional epidemiological studies.

Further investigations to confirm these associations and elucidate the underlying pathways are required. We evaluated a very limited number of bacteria and found *E. coli* and *S. pneumoniae* to have the strongest associations with gonococcal fluoroquinolone and macrolide resistance, respectively. It is possible that the associations may be stronger in other species. Crucially there is a need to monitor AMR in commensal *Neisseria* species in populations at high risk for gonococcal AMR [39, 40].

For both macrolides and fluoroquinolones, our results represent additional evidence for the community antibiotic theory. More specifically, they suggest that gonococcal antimicrobial resistance can be productively viewed as being part of a syndemic of resistance. The results, therefore, build on those from global analyses which found that a likely key reason why gonococcal AMR frequently emerged in core groups in Asia and elsewhere, was related to high consumption of antimicrobials in these populations [11, 40, 41]. ESCephalosporin resistance, for example, first emerged in Japan [19]. At least in part, this was likely due to the extraordinary high ESCephalosporin consumption in Japan at the time – over double the consumption of the country with the next highest consumption [11]. ESCephalosporin AMR emerged rapidly in other organisms in the Asia Pacific region around the same time but, as was the case with *N. gonorrhoeae*, it did not spread uniformly throughout the region [42–44]. In the rural parts of Northern Territory, Australia, for example, there is almost no gonococcal resistance to ESCephalosporins or azithromycin, a finding that is likely attributable to low antimicrobial consumption [43, 45]. Likewise, a phylogenetic analysis of 419 isolates from around the world found that modern gonococci were split into two lineages [41]. Lineage A was found to have arisen in Asia and had a high prevalence of AMR associated mutations (modal number of AMR mutations 7). This was thought to be due to high exposure to antimicrobials. The African lineage B, however, had far fewer AMR associated mutations (modal number of AMR mutations 0) – presumably due to lower antimicrobial exposure. These findings of populations with low levels of gonococcal AMR are important because they reveal that gonococcal AMR is not as inevitable as is commonly supposed but may be prevented or delayed [11, 46].

There are a number of important limitations to this analysis. The EARS-Net data are not based on uniform central testing of isolates. The breakpoints for resistance have also changed during the period under investigation. For example, EUCAST lowered its fluoroquinolone breakpoints for resistance in *K. pneumoniae* in 2016, which could influence longitudinal analyses such as the current one [28]. We did not adjust our analyses for either differences in MIC testing strategies or changes in breakpoints over time. In both cases, these factors would operate as misclassification biases which typically result in a bias towards the null hypothesis [47]. This would be expected to reduce the statistical strength of any association found. Our measure of antimicrobial consumption was based on ESAC data whose consumption estimates are very similar to those produced by different methodologies such as that used by IQVIA-MIDAS [48, 49]. We were unable to assess exposure using alternative measures such as days of therapy. The measure of consumption used does not, however, include consumption in hospitals which is an important determinant of AMR for some of the bacteria we evaluated. We were also unable to control for confounders, such as various environmental and socioeconomic variables that are associated with the spread of AMR [50]. It is also possible that STI services in high antimicrobial consumption countries may be more likely to prescribe antibiotics to clients than those in low consumption countries. Whilst we did not control for this in our models, it could be argued that it would be inappropriate to control for this since this is an effect mediator rather than a confounder. Finally, it is possible that a selection bias pertained whereby certain countries were more likely to send more resistant isolates for *N. gonorrhoeae* and the other bacteria. The fact that the *N. gonorrhoeae* AMR data is obtained from the Euro-GRASP survey, which has a separate surveillance system and does not depend on isolates from blood cultures makes this bias less likely. Validation studies have also concluded that, with the exception of beta-lactam resistance in *S. pneumoniae* and plasmid-mediated colistin resistance in the *Enterobacteriaceae*, the EARS-net AMR prevalence estimates are relatively accurate [51].

Evidence that gonococcal AMR is part of a syndemic of resistance is an important finding as it suggests that minimizing the probability of further AMR emerging in *N. gonorrhoeae* would benefit from antibiotic stewardship campaigns to reduce total consumption of antibiotics. The probability of bystander selection affecting *N. gonorrhoeae* is considerably higher in core-groups such as HIV preexposure prophylaxis populations where the prevalence of *N. gonorrhoeae* is around 10% [52, 53]. Gonococcal AMR has also frequently emerged in these types of core groups with high gonococcal prevalence and high antimicrobial consumption [40, 53]. As a result, it may be prudent to focus stewardship campaigns on both general populations with high antimicrobial consumption as well as core-groups in all populations [54].

## Supplementary information

**Additional file 1 Figure S1.** Fluoroquinolone (FQ) consumption and prevalence of antimicrobial resistance to fluoroquinolones in *Neisseria gonorrhoeae* (Ng) and *Escherichia coli* (Ec) in 30 European countries. **Figure S2.** Macrolide consumption and prevalence of antimicrobial resistance to azithromycin in *Neisseria gonorrhoeae* (Ng) and macrolides in *Streptococcus pneumoniae* (Sp) in 30 European countries.

## Data Availability

The data we used is publicly available from: https://atlas.ecdc.europa.eu/
