# Supplementary material for: Gonococcal resistance can be viewed productively as part of a syndemic of antimicrobial resistance: an ecological analysis of 30 European countries
Source: Antimicrob Resist Infect Control. 2020 Jun 30;9:97. doi: 10.1186/s13756-020-00764-z (PMC7325135; doi:10.1186/s13756-020-00764-z)
Supplement: Supplementary file 1 — Additional file 1 Figure S1. Fluoroquinolone (FQ) consumption and prevalence of antimicrobial resistance to fluoroquinolones in Neisseria gonorrhoeae (Ng) and Escherichia coli (Ec) in 30 European countries. Figure S2. Macrolide consumption and prevalence of antimicrobial resistance to azithromycin in Neisseria gonorrhoeae (Ng) and macrolides in Streptococcus pneumoniae (Sp) in 30 European countries. [file 13756_2020_764_MOESM1_ESM.docx]

**Online Supplementary File**

**Contents**

[Figure S1: Fluoroquinolone (FQ) consumption and prevalence of antimicrobial resistance to fluoroquinolones in *Neisseria gonorrhoeae* (Ng) and *Escherichia coli* (Ec) in 30 European countries 2](#_Toc32599900)

[Figure S2: Macrolide consumption and prevalence of antimicrobial resistance to azithromycin in *Neisseria gonorrhoeae* (Ng) and macrolides in Streptococcuc pneumoniae (Sp) in 30 European countries 3](#_Toc32599901)

# Figure S1: Fluoroquinolone (FQ) consumption and prevalence of antimicrobial resistance to fluoroquinolones in *Neisseria gonorrhoeae* (Ng) and *Escherichia coli* (Ec) in 30 European countries

Abbreviations: AT, Austria; BE, Belgium; CZ, Czech Republic; DE, Germany; DK, Denmark; EE, Estonia; EL, Greece; ES, Spain; FI, Finland; FR, France; HR, Croatia; HU, Hungary; IE, Ireland; IT, Italy; LU, Luxembourg; LV, Latvia; NL, the Netherlands; NO, Norway; PL, Poland; PT, Portugal; SE, Sweden; SI, Slovenia; SK, Slovakia; UK, United Kingdom.

# Figure S2: Macrolide consumption and prevalence of antimicrobial resistance to azithromycin in *Neisseria gonorrhoeae* (Ng) and macrolides in *Streptococcus pneumoniae* (Sp) in 30 European countries

Abbreviations: AT, Austria; BE, Belgium; CZ, Czech Republic; DE, Germany; DK, Denmark; EE, Estonia; EL, Greece; ES, Spain; FI, Finland; FR, France; HR, Croatia; HU, Hungary; IE, Ireland; IT, Italy; LU, Luxembourg; LV, Latvia; NL, the Netherlands; NO, Norway; PL, Poland; PT, Portugal; SE, Sweden; SI, Slovenia; SK, Slovakia; UK, United Kingdom.
